# Konjac Glucomannan: From Molecular Architecture to Translational Applications—A Critical Review of Structure–Function Relationships, Emerging Biomedical Frontiers, and Industrial Challenges

**DOI:** 10.3390/foods15162872

**Published:** 2026-08-17

**Authors:** Wencai Bai, Mengjie Li, Bo Ding, Min Hu, Yongqiang An, Haoyang Xie, Qingyu Wen, Lishui Chen, Liang Zhao

**Affiliations:** 1 Food Laboratory of Zhongyuan, Henan University of Technology, Zhengzhou 450001, China; 2 College of Food Science and Nutritional Engineering, China Agricultural University, Beijing 100083, China

**Keywords:** konjac glucomannan, structure–function relationship, drug delivery, wound healing, gut microbiota, biopolymer, polysaccharide, biomaterial

## Abstract

Konjac glucomannan (KGM) is a plant-derived polysaccharide with a long history of food use and a rapidly expanding portfolio of biomedical applications. Yet despite decades of research, translation of KGM-based materials from laboratory proof-of-concept to clinical and commercial products remains slow, hampered by unresolved structural controversies, batch-to-batch variability, and a lack of quantitative design rules. This review provides a critical, mechanism-focused analysis of KGM across its molecular architecture, extraction and modification, and translational applications, moving beyond cataloguing uses to evaluate conflicting findings in the literature. We examine how hierarchical structural features—molecular weight, acetylation pattern, and chain topology—govern solution behavior, gelation, and biological performance, and compare KGM with competing biopolymers to define its unique advantages and inherent limitations. Current progress in colon-targeted delivery, wound healing, microbiome modulation, and metabolic health is synthesized, and persistent barriers to translation are evaluated, including the absence of quantitative structure–activity models, limited human pharmacokinetic data, and incomplete toxicological characterization of modified derivatives. We also address longstanding debates over branching frequency, acetylation distribution, and dose–response relationships that have generated inconsistent results across studies. Finally, we outline a research agenda integrating computational polymer design, multi-omics mechanistic studies, and precision nutrition to support development of next-generation KGM biomaterials.

## 1. Introduction

Growing demand for sustainable, biocompatible materials has renewed research interest in natural polysaccharides. KGM is a plant-derived natural polysaccharide with unique functional properties, including high water-holding capacity, alkali-induced thermoirreversible gelation, and specific susceptibility to colonic β-mannanase, which distinguish it from most other common food gums [1,2]. Annual global production of purified KGM exceeds 30,000 metric tons, with applications spanning traditional Asian foods, pharmaceutical excipients, and next-generation biomedical devices [3].

More than four decades of research have yielded a large body of data on KGM, but several fundamental questions remain unresolved. The influence of acetylation on gelation is well established at a qualitative level, but the topological distribution of acetyl groups along the polymer chain and its quantitative effect on junction zone formation remain debated [1,4]. Similarly, while KGM is widely regarded as a prebiotic, the primary degraders in the human gut microbiota and the cross-feeding networks underlying its metabolic effects have only begun to be clarified using multi-omics approaches [2]. A persistent translational gap separates lab-scale KGM delivery systems from clinically available commercial products, alongside challenges such as unstable batch performance of raw polysaccharide, insufficient toxicological characterization for chemically modified derivatives, limited human pharmacokinetic evidence, and low feasibility of scaling most lab-level fabrication technologies [5].

Previous reviews have generally adopted an application-focused approach, describing uses of KGM in foods, pharmaceuticals, and biomaterials without critical evaluation of conflicting findings or detailed mechanistic discussion [3,6]. This work frames the literature through a hierarchical structure–function lens, linking molecular architecture to macroscopic performance, and provides a head-to-head comparison of KGM with competing biopolymers to clarify where its genuine advantages lie. Longstanding controversies that have generated inconsistent results are addressed explicitly, and specific high-priority research gaps are identified. A convergent research roadmap integrating artificial intelligence, synthetic biology, and precision medicine is proposed to guide future KGM research over the next decade.

## 2. Molecular Architecture and Hierarchical Structure–Function Relationships

### 2.1. Primary Structure: Sequence, Substitution Pattern, and Molecular Weight

**Figure 1:**
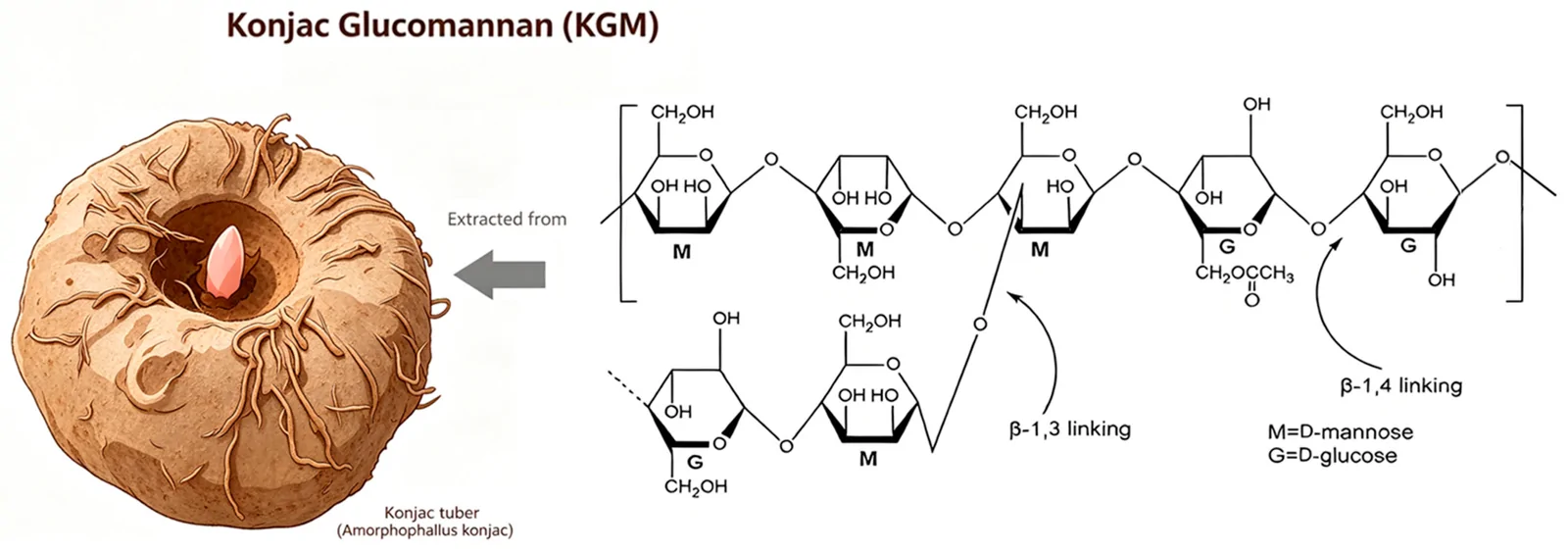
Structure of native KGM.

KGM is a linear polysaccharide consisting of β-D-glucose (Glc) and β-D-mannose (Man) residues linked by (1 → 4) glycosidic bonds. The Man:Glc ratio typically ranges from 1.4:1 to 1.8:1, depending on the plant species and extraction method used [1]. A key structural feature is the presence of O-acetyl groups substituted at the C-2, C-3, and/or C-6 positions of Glc and Man residues, with an average degree of substitution (DS) of approximately one acetyl group per 9–19 sugar residues (Figure 1). Functionally, these acetyl groups do not merely improve solubility; they act as molecular spacers that disrupt interchain hydrogen bonding and shape the final gel network architecture. Recent molecular dynamics simulations by Sun et al. demonstrated that uniformly distributed acetyl groups promote formation of dense, homogeneous aggregates, whereas clustered acetylation leads to heterogeneous, weak networks—a finding with direct implications for quality control of commercial KGM grades [4].

Beyond acetylation, another debated structural feature of KGM is its degree of branching. The longstanding discrepancy between early methylation analyses reporting 5–8% branching and modern structural studies indicating a substantially lower degree of branching appears to arise primarily from sample preparation artifacts [7]. Early per-methylation studies employing GC–MS identified 2,6-di-O-methyl derivatives of both D-mannose and D-glucose, confirming branch points at C-3 of both sugar residues in the β-1,4-linked backbone, with branches occurring more frequently at mannose than at glucose residues [7]. However, these investigations often suffered from incomplete polysaccharide dissolution, leaving aggregated chain segments that were likely misinterpreted as covalent branches during linkage analysis. It is now recognized that KGM does not form true molecular solutions but rather exists as dispersions containing persistent micro-aggregates—even after prolonged stirring and heating—due to the “fish-eye” effect, where rapidly hydrated surface layers delay further swelling and disentanglement of the polymer core. Contemporary structural characterizations describe KGM as possessing only a small degree of β-1,3-branching predominantly at the C-3 position of mannosyl residues, with a few additional branches potentially linked at O-2,6 of mannosyl units and/or O-3,6 of glucosyl units [8]. The inherently poor solubility of KGM across various solvent systems continues to present methodological challenges for accurate structural analysis, as incomplete solubilization can compromise both chemical modification and spectroscopic measurements [9]. Furthermore, branching frequency may vary significantly among KGM from different Amorphophallus species and isolation protocols, and this source of structural variability has not yet been systematically investigated [7].

The molecular weight (Mw) of commercial KGM ranges from 200 kDa to over 2000 kDa, with polydispersity indices typically between 1.3 and 2.0. Li et al. showed that Mw is the primary parameter governing both rheological properties and digestive behavior: high-Mw KGM (>1000 kDa) exhibits 3–5-fold higher viscosity and significantly greater postprandial glucose-lowering effects than low-Mw fractions (<500 kDa) [10].

### 2.2. Hierarchical Assembly: From Helix Formation to Gel Network

**Figure 2:**
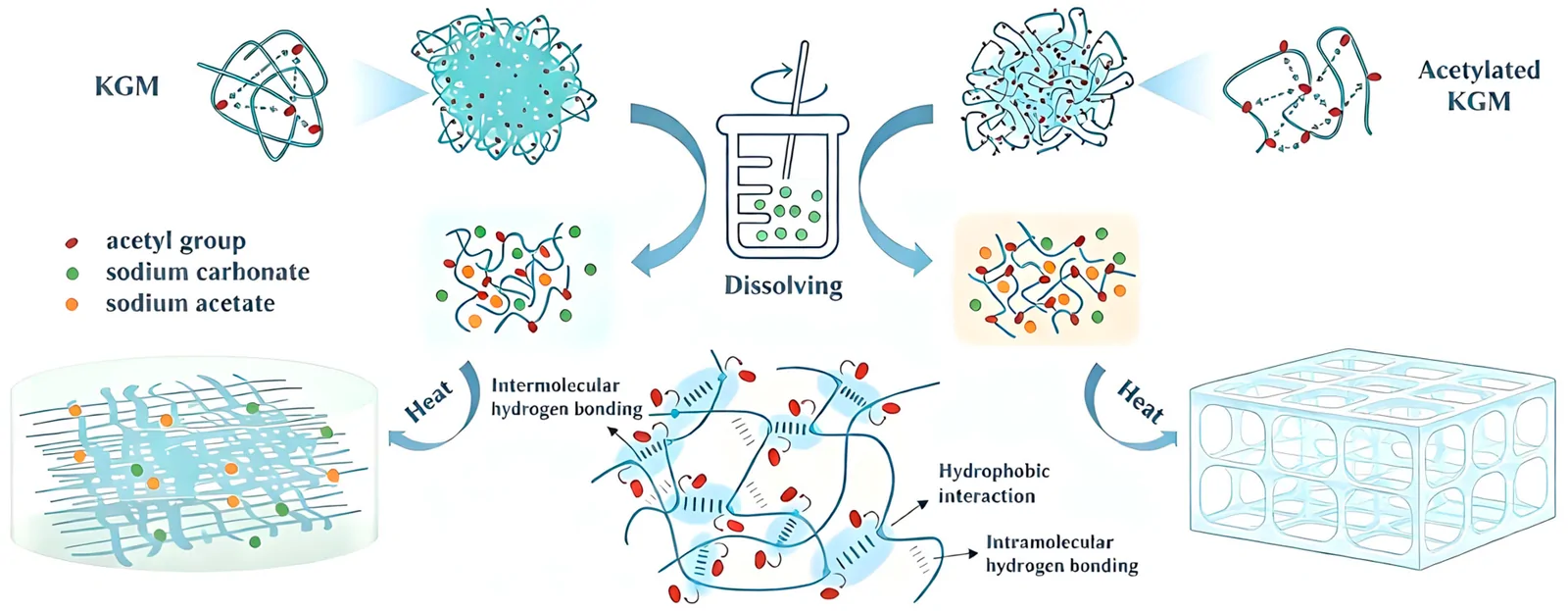
Schematic illustration of structural evolution and acetyl-mediated gelation mechanism of KGM. Adapted from [1].

The aqueous solubility of KGM is highly dependent on molecular weight and temperature. Typical commercial KGM with molecular weight of 500–1000 kDa can be fully dispersed in cold water at concentrations up to 1–2 wt%, while higher temperature markedly improves hydration efficiency. Low-molecular-weight KGM fractions exhibit superior dispersibility, whereas high-molecular aggregates tend to form undissolved lumps and require extended stirring or heating for full hydration [8]. In aqueous solution, KGM chains adopt an extended, disordered conformation with minimal helical character, in contrast to helix-forming polysaccharides such as carrageenan and gellan gum. Upon alkali-induced deacetylation (typically pH > 9, temperature > 70 °C), KGM undergoes a sol–gel transition to form thermoirreversible gels, a property uncommon among common neutral polysaccharides (Figure 2) [11]. The gelation process proceeds through saponification of acetyl groups, which exposes previously shielded hydroxyl groups along the chain. Trace deacetylated segments then act as nucleation sites, followed by cooperative growth of interchain hydrogen-bonded junction zones into a three-dimensional network [1]. This gelation does not follow a continuous second-order transition but exhibits sharp, first-order-like cooperative behavior, consistent with a nucleation–growth mechanism.

KGM generates strong synergistic interactions with various polysaccharides for industrial gel production. For the 1:1 mass blending system of KGM and κ-carrageenan, significant synergistic elevation of gel hardness is achieved via intermolecular forces between KGM mannose units and κ-carrageenan double helices. Deacetylation of KGM further amplifies this synergy by facilitating the formation of more hydrogen bonds and hydrophobic crosslinks within the composite network [12]. KGM blended with xanthan gum forms thermoreversible synergistic interaction gels. Cyclic directional freeze–thaw (DFT) treatment aligns polymer microdomains, which nearly doubles the average storage modulus of K4X6 gel from around 100 Pa to approximately 200 Pa [13]. These effects are not merely additive but represent emergent properties arising from specific intermolecular interactions that remain poorly characterized at the atomic level.

Beyond gel network formation, the conformational flexibility and hydration behavior of KGM chains also give rise to a range of stimuli-responsive physicochemical properties. pH-responsive swelling arises from the protonation and deprotonation of hydroxyl groups along the KGM backbone, which modulates electrostatic repulsion between chains and shifts the balance between interchain hydrogen bonding and water uptake. Temperature-dependent swelling behavior is intrinsically linked to the thermodynamics of KGM-water interactions: elevated temperatures weaken hydrogen bonding between KGM chains and water molecules, altering chain hydration state and network water retention capacity, an effect that can be further modulated by varying the degree of acetylation. These stimuli-responsive properties enable the design of smart delivery systems with triggered release capabilities.

### 2.3. Comparative Advantage: KGM Versus Competing Biopolymers

To contextualize KGM among natural biopolymers, Table 1 systematically compares it with alginate, chitosan, xanthan gum, and gellan gum across key performance metrics relevant to food and biomedical applications. This comparison shows that KGM occupies a distinct intermediate position: it lacks the cationic charge of chitosan (advantageous for mucoadhesion but potentially cytotoxic at high molecular weights) and the ionic gelation capacity of alginate (convenient for encapsulation but sensitive to physiological calcium concentrations), but offers high swelling capacity, specific colonic degradability, and generally recognized as safe (GRAS) status for oral consumption.

KGM is the only commercially available polysaccharide that combines enzyme-specific colon targeting, thermoirreversible gelation without toxic crosslinkers, and established dietary fiber health claims. This combination makes it particularly well suited for oral drug delivery and functional food applications.

## 3. Extraction, Purification, and Modification: Critical Assessment of Industrial Readiness

### 3.1. Dry Versus Wet Extraction: A Trade-Off Between Cost and Quality

Industrial KGM is produced via two distinct approaches that yield materials with different properties and suitability for different applications. Dry extraction is the dominant traditional method. It uses mechanical disruption and air classification to separate KGM vesicles (100–500 μm) from starch and fiber without solvents [20]. This process yields KGM with 60–80% purity at low cost, but has two inherent limitations. Mechanical shear during milling causes substantial Mw degradation, typically a 20–40% reduction. Small-molecule impurities—including oxalate crystals, residual protein, pigments, and trimethylamine (which causes a fishy off-flavor)—remain trapped within vesicle matrices. The resulting material is suitable for general food use but not for pharmaceutical or biomedical applications.

Wet extraction, which involves water solubilization followed by ethanol precipitation, produces high-purity KGM (85–97%) with preserved Mw and improved color and odor profiles [20]. Conventional wet extraction consumes large volumes of water and ethanol (typically 20–50 volumes of ethanol per unit mass of KGM), increasing both cost and environmental impact.

Emerging green extraction technologies—including enzyme-assisted, ultrasound-assisted, and high-pressure homogenization methods—have achieved 10–25% yield improvements at laboratory scale, but significant scale-up challenges remain. Most laboratory studies of enzyme-assisted extraction report yield gains based on small-batch experiments with well-controlled temperature and pH; few address the mass transfer limitations that arise in scale-up, where uneven enzyme distribution and prolonged reaction times can lead to uncontrolled molecular weight degradation. Ultrasonic extraction suffers from similar issues, with uneven energy distribution in industrial-scale vessels leading to variable Mw degradation across batches [21]. Chen et al. recently reported a reagent-assisted heterogeneous hygrothermal degradation method that shows promise for controlled production of low-Mw KGM, but its industrial feasibility has not yet been demonstrated [22].

### 3.2. Chemical Modification: Rational Design Versus Trial-and-Error

Native KGM has several drawbacks that motivate chemical modification: poor room-temperature solubility at concentrations above 2%, rapid syneresis during freeze–thaw cycles, limited hydrogel mechanical strength, and burst release when used as a drug carrier [23]. Four major modification strategies have been explored: carboxymethylation, oxidation, sulfation, and graft copolymerization.

A critical observation from the KGM structural characterization literature is that most studies remain descriptive and comparative rather than rationally designed, with limited systematic investigation of structure–property relationships [24]. Most investigations report basic compositional parameters—such as molecular weight, monosaccharide ratio, and degree of acetylation—alongside conventional rheological measurements, yet few studies systematically quantify how specific structural features govern functional performance or resolve fine structural details such as substitution distribution along the chain [25]. This gap is significant because, as is well established in the modified starch industry, the spatial pattern of substituent groups (e.g., clustered vs. uniform distribution along the backbone) can exert a far greater influence on solution and gel properties than the average degree of substitution alone—a principle that has driven decades of starch modification research but has rarely been applied to KGM derivatization [26]. Furthermore, the toxicological profile of most chemically modified KGM derivatives remains inadequately characterized. While native KGM is generally recognized as safe (GRAS) and has a long history of food use, chemical modification introduces new functional groups whose metabolic fate, potential immunogenicity, and long-term safety have not been systematically evaluated. This represents a major, underappreciated translational barrier that must be addressed before modified KGM derivatives can advance to clinical or biomedical applications [27].

## 4. Pharmaceutical and Biomedical Applications: Mechanisms, Advances, and Translational Barriers

### 4.1. Colon-Targeted Drug Delivery: Exploiting Enzyme Specificity

A key pharmaceutical property of KGM is its selective degradation by β-mannanase produced by colonic *Bacteroides* and *Bifidobacterium* species, combined with resistance to upper gastrointestinal amylases and proteases [2]. This enzyme-specific trigger provides intrinsic colon-targeting capability without the need for pH-dependent coatings or time-dependent release systems, an advantage that has been widely exploited for inflammatory bowel disease (IBD) and colorectal cancer therapy. Zhou et al. developed a KGM/sodium alginate/ε-poly-L-lysine hydrogel that forms in situ on mucosal surfaces via hydrogen bonding and electrostatic interactions, demonstrating both esophageal and colonic wound healing efficacy through epithelial proliferation and inflammatory modulation [28]. Liang et al. applied this approach to Pickering emulsion gels stabilized by sardine protein/KGM complexes for astaxanthin delivery in IBD. They found that KGM significantly improved both emulsion stability and in vivo therapeutic efficacy compared with protein-only systems [29].

Even so, several translational barriers remain. The extent of KGM degradation varies substantially between individuals due to differences in gut microbiota composition. This is particularly relevant for patients with IBD, whose microbiomes are often dysbiotic with reduced bacteroides abundance. Most studies use single-pathogen or chemical-induced colitis models that do not fully recapitulate the complexity of human IBD, particularly its chronic, relapsing nature. The mucoadhesive properties of KGM, while generally advantageous, may lead to variable transit times and release kinetics in vivo. Future systems should incorporate microbiome-agnostic release triggers (e.g., redox potential, specific enzyme combinations) alongside enzyme-responsive degradation of KGM to improve inter-patient reliability.

### 4.2. Probiotic and Bioactive Delivery: Protecting Viability Through the GI Tract

The protective effect of KGM matrices on probiotic viability during gastric and intestinal transit has been demonstrated in multiple encapsulation systems. Chen et al. reported that alginate/KGM composite beads at a 1:3 weigh ratio achieved 81.5% encapsulation efficiency for *Lactobacillus plantarum*, with significantly enhanced survival during cold storage and simulated gastrointestinal digestion compared to pure alginate beads [19]. Guo et al. extended this work to W/O/W double emulsions stabilized by whey protein isolate–KGM non-covalent complexes, achieving high encapsulation efficiency and improved viability after pasteurization [30]. The protective mechanism appears to involve two effects: the high viscosity of KGM reduces molecular mobility and oxygen permeability, and its buffering capacity partially neutralizes gastric acid penetration.

**Figure 3:**
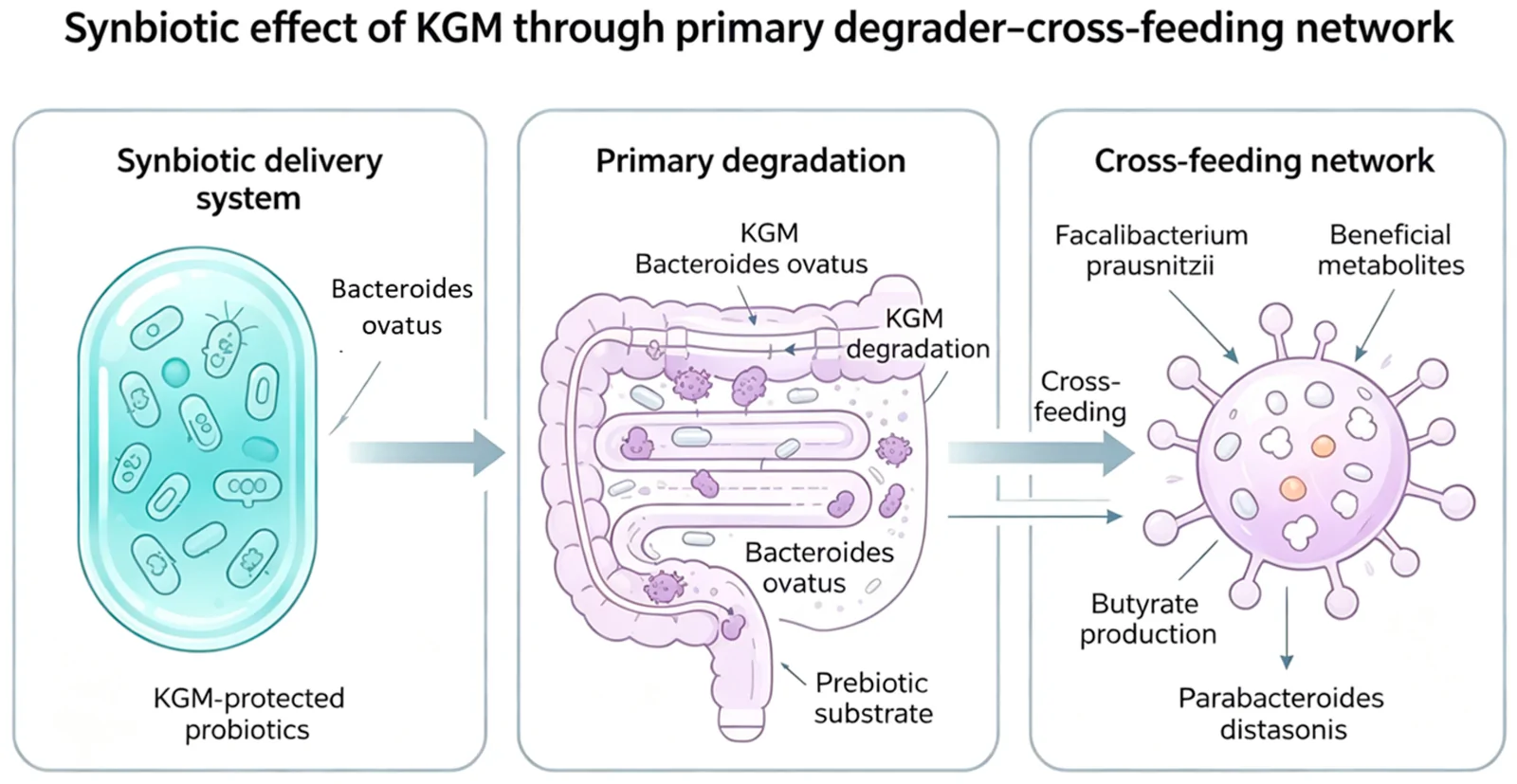
In vitro human gut microbiota fermentation of KGM and corresponding fermentation characterization. Adapted from [2].

An important but understudied aspect is the synbiotic effect: KGM not only protects probiotics during delivery but also acts as a selective prebiotic substrate that promotes the growth and metabolic activity of probiotics once released in the colon. Using multi-omics analysis, Sun et al. recently showed that KGM is primarily degraded by *Bacteroides ovatus*, which cross-feeds beneficial butyrate producers including *Faecalibacterium prausnitzii* and *Parabacteroides distasonis* [2] (Figure 3). This primary degrader–cross-feeding network provides a mechanistic basis for the prebiotic effects of KGM, and suggests that rational synbiotic combinations—KGM plus specific primary degraders and butyrate producers—may outperform KGM alone.

### 4.3. Wound Dressings and Tissue Engineering Scaffolds

KGM-based hydrogels are promising wound dressing materials due to their high water retention, biocompatibility, and intrinsic hemostatic properties. Li et al. developed silver nanoparticle-loaded KGM/silk fibroin composite hydrogels that combined the antibacterial properties of AgNPs with the moist healing environment provided by KGM, demonstrating accelerated wound closure, enhanced angiogenesis, and increased collagen deposition in a full-thickness skin defect model [31]. Hao et al. adopted an herbal medicine approach by co-crosslinking KGM with *Bletilla striata* polysaccharide, creating a composite hydrogel that inhibited the TNF-α/NF-κB inflammatory pathway and promoted cell migration and wound closure in mice [14]. In an innovative approach, Zhong et al. developed lipoic acid-modified KGM hydrogels loaded with siACTC1-exosomes for post-surgical keloid treatment. These hydrogels achieved sustained ACTC1 inhibition and reduced mechanical tension in a mouse model [32].

**Figure 4:**
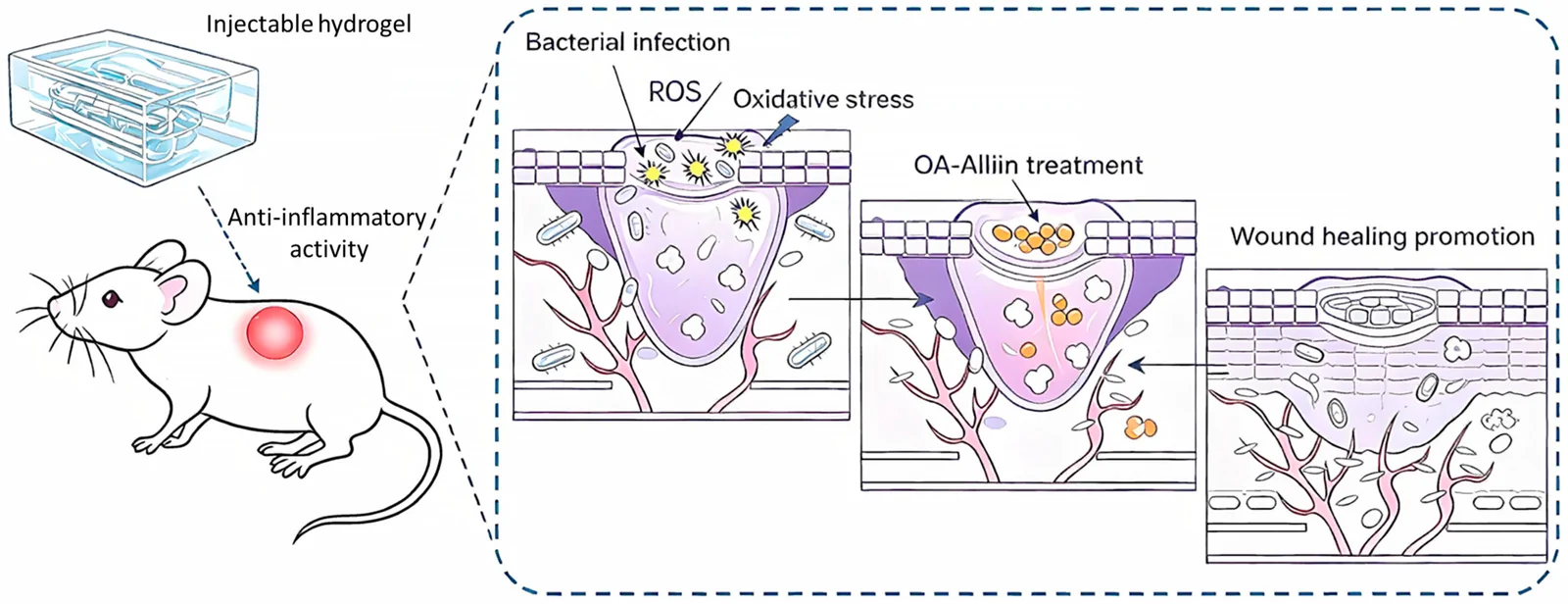
Fabrication and in vivo wound healing performance of self-healing injectable OKGM/AG/allicin hydrogel. Abbreviations: OKGM, oxidized konjac glucomannan; AG, aminated gelatin; OA-Allicin, allicin-loaded composite hydrogel consisting of oxidized konjac glucomannan and aminated gelatin. Adapted from [34].

Carboxymethylated KGM (CMKGM) is the most widely studied derivative, with improved cold-water solubility and pH-responsive swelling. Its degree of substitution is difficult to control precisely, and over-substitution (DS > 0.8) completely eliminates gelation capacity [33]. Oxidized KGM (OKGM), typically prepared using TEMPO/NaBr/NaOCl or H_2_O_2_ systems, introduces aldehyde and carboxylate groups that enable Schiff-base crosslinking with amino-containing polymers such as chitosan, gelatin, and proteins. This crosslinking forms self-healing, injectable hydrogels that are of interest for wound dressings and cell delivery [34] (Figure 4).

In tissue engineering, KGM has been explored for skin, bone, cartilage, and even muscle tissue scaffolds. Shao et al. demonstrated that incorporation of KGM enhanced the compressive strength of polyacrylamide hydrogels from 140.78 kPa to 638.79 kPa while reducing friction coefficients by >50%, a finding relevant for cartilage replacement [17]. Pietri et al. reported a textile-inspired wet-spinning approach to produce KGM monofilaments at 15 m/min that supported attachment and differentiation of C2C12 myoblasts, offering a cost-effective scaffold for cultivated meat production [35]. KGM scaffolds have lower mechanical strength and less tunable degradation than synthetic polymers such as PLA (poly(lactic acid)) and PCL (poly(ε-caprolactone)), and no KGM-based tissue engineering product has yet received regulatory approval for human clinical use.

### 4.4. Emerging Smart Delivery Systems: pH-, Thermo-, and Redox-Responsive Platforms

Building on the stimuli-responsive conformational and swelling properties of KGM, smart delivery systems that exploit pH-, temperature-, and redox-triggered release have emerged as a rapidly growing area of research. Ding et al. developed pH/temperature dual-responsive hydrogels composed of KGM and poly(N-isopropylacrylamide-co-crotonic acid) (denoted P(NIPAM-co-CA)), where CA stands for crotonic acid, NIPAM is N-isopropylacrylamide. N,N′-methylenebisacrylamide (MBA) acts as the crosslinker, while ammonium persulfate (APS) and tetramethylethylenediamine (TEMED) serve as the initiator pair during copolymerization. This composite hydrogel achieved a 1.8-fold higher cumulative release of 5-fluorouracil in simulated colonic fluid compared to simulated gastric fluid, verifying its capability for programmable colon-targeted drug delivery [36]. Cui et al. prepared acetylated KGM nanoparticles for ACE inhibitory peptide delivery, achieving 88.37% encapsulation efficiency with sustained release under simulated gastrointestinal conditions [37]. Basification of KGM aerogels via supercritical CO_2_ drying has enabled high-load delivery of lipophilic bioactives such as β-carotene, addressing the inherent hydrophilicity limitation of KGM [16]. Most smart KGM systems have only been characterized in vitro; pharmacokinetic data from animal models, let alone humans, remain very limited.

## 5. Biomedical Health Benefits: From Phenomenology to Mechanistic Understanding

While Section 4 uses physicochemical traits of KGM (gelation, enzymatic suscepti-bility, mucoadhesion) to fabricate biomaterials for drug delivery and wound repair, this section covers health effects of oral KGM on metabolic and intestinal disorders. Two linked pathways mediate these outcomes: viscosity-based regulation in the upper gut, and colonic microbial fermentation that produces short-chain fatty acids to remodel host metabolism.

### 5.1. Gut Microbiota Modulation: Decoding the Primary Degrader–Cross-Feeding Network

The prebiotic effects of KGM are among its most studied health benefits, and mechanistic understanding has advanced substantially in the past three years through multi-omics approaches. Wang et al. identified a degree-of-acetylation-dependent effect: higher acetylation enhanced the ability of KGM to enrich *Bacteroides uniformis* and improve glucose and lipid metabolism in prediabetic mice [38]. Sun et al. identified *Bacteroides ovatus* as the primary KGM degrader in human fecal microbiota, demonstrating that this species possesses specific polysaccharide utilization loci (PULs) for KGM breakdown and that degradation products cross-feed beneficial species including *F. prausnitzii* and *P. distasonis* [2]. This observation revisits the current understanding of KGM-mediated prebiotic activity, moving beyond the oversimplified paradigm that KGM exclusively stimulates bifidobacterial populations toward a refined ecological framework encompassing specialized primary degraders and interconnected cross-feeding cascades.

Clinical evidence supporting the beneficial effects of konjac glucomannan (KGM) on gut health is available from a double-blind randomized controlled trial (RCT) performed by Zhu et al. [39]. The study enrolled elite athletes diagnosed with functional constipation, a population that exhibits a particularly high prevalence of constipation due to chronic dehydration, restrictive dietary patterns, and intensive exercise-induced stress. In this trial, KGM supplementation yielded significant improvements in constipation symptoms, as validated by evaluations using the Patient Assessment of Constipation-Symptoms (PAC-SYM) scale for symptom severity and the Patient Assessment of Constipation-Quality of Life (PAC-QoL) scale for life quality. Additionally, the intervention elevated the abundance of prevotella and *Lactobacillus* in the gut microbiota. The laxative properties of KGM can be explained by two synergistic mechanisms. The strong water-holding capacity of KGM directly elevates fecal water content and increases stool bulk. Meanwhile, the production of short-chain fatty acids mediated by Prevotella stimulates colonic peristalsis, thereby facilitating intestinal transit [39]. Most prebiotic studies have been conducted in healthy individuals or animal models. The effects of KGM in dysbiotic states such as IBD, IBS, or obesity may differ substantially due to altered baseline microbiota composition, and this remains an important area for future clinical research.

### 5.2. Metabolic Health: Weight Management, Glycemic Control, and Lipid Lowering

The metabolic benefits of KGM—including weight reduction, improved glycemic control, and cholesterol lowering—are supported by both mechanistic studies and clinical trials. A comprehensive meta-analysis of RCTs published between 2014 and 2024 by Ghosh et al. found that KGM supplementation (≥5 g/day for ≥12 weeks) was associated with significant reductions in BMI (weighted mean difference, WMD: −1.49 kg/m^2^), body weight (WMD: −3.18 kg), and waist circumference (WMD: −2.11 cm) in overweight and obese subjects. These effects are mediated by viscosity-induced satiety, delayed gastric emptying, and microbiome modulation—specifically enrichment of *Bacteroidetes*, *Akkermansia*, and *Bifidobacterium*, with increased short-chain fatty acid (SCFA) production [40].

For glycemic control, Dehzad et al. showed in an RCT that yogurt fortified with KGM and inulin for 8 weeks significantly improved fasting insulin (−1.85 µIU/mL), HOMA-IR (−0.89), and QUICKI (+0.11) in patients with type 2 diabetes, accompanied by reduced total cholesterol (−18.51 mg/dL) and triglycerides (−15.0 mg/dL) [41]. Jin et al. recently elucidated the cholesterol-lowering mechanism, showing that high-Mw KGM inhibits intestinal fatty acid uptake more effectively than degraded oligosaccharide products, by enriching *Lactobacillus*, *Desulfovibrio*, and *Allobaculum* while enhancing intestinal barrier function [42]. Xu et al. showed that KGM-embedded resistant starch produced significantly lower postprandial blood glucose peaks (7.08 versus 8.57 mmol/L) in mice, demonstrating a food-matrix approach to glycemic management [43].

### 5.3. Anti-Tumor and Immunomodulatory Activities: Promise Versus Proof

Reports of direct anti-tumor activity of KGM should be interpreted with considerable caution. Most studies claiming anti-cancer effects have used either sulfated/oxidized KGM derivatives (not native KGM) or KGM as a delivery vehicle for established anti-cancer agents, rather than demonstrating direct tumoricidal effects of KGM itself. The antioxidant and anti-inflammatory activities are better substantiated: sulfated KGM fragments prepared via relay strategy showed strong free radical scavenging and protected RAW264.7 cells against H_2_O_2_-induced injury through Nrf2/Keap1 pathway activation [44]. Wu et al. developed zinc-doped carbon dot/quaternized chitosan/tannic acid/KGM composite films with ~90% free radical scavenging and broad-spectrum antibacterial properties [15]. Direct systemic immunomodulatory effects of orally administered native KGM—beyond those mediated by SCFAs from microbial fermentation—remain speculative and require rigorous investigation in adequately powered human studies.

## 6. Controversies and Unresolved Debates in KGM Research

Despite decades of research on KGM, several longstanding controversies remain unresolved, leading to inconsistent conclusions and poor comparability across published studies. These discrepancies arise primarily from differences in sample preparation protocols, analytical methods, and parameter reporting standards. Three core debates that limit the development of a unified structure–function framework are outlined below.

Branching frequency: The discrepancy between early methylation analysis studies reporting 5–8% branching and modern NMR/SAXS studies reporting <1% branching appears to arise primarily from sample preparation artifacts. Early studies often used incomplete dissolution conditions, leaving aggregated chain segments that were misinterpreted as covalent branches. It remains possible that branching varies significantly between KGM from different amorphophallus species and different extraction methods, and this source of structural variability has not been systematically investigated.

Acetylation: degree versus distribution pattern: Most studies report only the average degree of acetylation, but recent simulation work suggests that the spatial distribution of acetyl groups (uniform versus clustered) may be more important than average DS for gelation behavior. Experimental validation of this hypothesis using precisely synthesized KGM standards with defined acetylation patterns has not yet been performed, representing a major gap in structure–function understanding.

Dose–response relationships for metabolic effects: Human clinical trials have implemented daily KGM supplementation doses spanning 1 g to 15 g, yet a unified quantitative dose–response profile for cholesterol reduction, glycaemic regulation, and weight management has not been validated across available clinical evidence [40]. Multiple controlled trials have observed meaningful metabolic improvements at a daily intake of 3 g KGM, matching the official EFSA health claim threshold for weight-lowering effects under energy-restricted diets [18]. Conversely, a subset of human interventions fails to detect statistically significant metabolic benefits when daily supplementation doses stay below 10 g [40]. Such inconsistent clinical outcomes are primarily attributed to variable intrinsic properties of KGM raw materials (including molecular weight and hydration capacity), diverse food delivery matrices, and interindividual disparities in baseline gut microbiota profiles. To date, the independent quantitative contribution of each confounding factor to divergent trial outcomes remains uncharacterized [40].

## 7. Safety, Regulation, and Translational Gap Analysis

### 7.1. Established Safety Profile of Native KGM

Native KGM has an established safety profile, with FDA GRAS status (21 CFR §184.1324) and EFSA approval as a food additive (E425). Acute toxicity studies report an LD_50_ > 10 g/kg in rodents, and 90-day subchronic feeding studies identified a no-observed-adverse-effect level (NOAEL) of >2.5 g/kg/day with no evidence of organ toxicity or genotoxicity [6]. The EFSA Panel on Dietetic Products has approved a health claim stating that daily consumption of 3 g of KGM contributes to the maintenance of normal blood cholesterol levels [18].

Safety concerns associated with KGM relate primarily to its physical properties and impurity profile. Esophageal or intestinal obstruction can occur if dry powder or tablet forms are taken with insufficient fluid, a risk that has prompted FDA choking hazard warnings. Gastrointestinal discomfort, including bloating, flatulence, and loose stools, may occur at daily doses above 10 g as a result of rapid colonic fermentation. Crude KGM preparations also contain calcium oxalate crystals, which may present a risk for individuals with a history of kidney stones [6].

### 7.2. The Translational Gap: Why So Few Products Despite So Many Publications?

The slow translation of KGM-based biomaterials reflects a combination of regulatory, technical, and commercial barriers. Chemically modified KGM derivatives do not inherit the GRAS status of the native polymer; each new derivative requires independent toxicological evaluation for its intended use, imposing costs and timelines that are rarely addressed in academic studies. Batch-to-batch variability in raw material properties—including molecular weight, acetylation degree, and impurity levels—further complicates translation, as pharmaceutical manufacturing requires consistent performance to meet GMP standards.

KGM also remains less established in pharmaceutical formulation than conventional excipients such as hydroxypropyl methylcellulose, alginate, and chitosan, despite its unique colon-targeting properties. This limited adoption reduces industry familiarity and slows uptake of new KGM-based systems. Many published fabrication and modification protocols rely on organic solvents, specialized equipment, or multi-step processing that is not economically feasible at industrial scale, creating a gap between laboratory demonstration and manufacturability.

## 8. Critical Research Gaps and a Convergent Future Roadmap

### 8.1. Identified High-Priority Research Gaps

Several interconnected research gaps currently limit the translational development of KGM. Most fundamentally, there is a lack of quantitative structure–activity relationship (QSAR) models that can reliably link structural features—molecular weight, acetylation degree and distribution, branching, and substituent pattern—to functional outcomes such as gel mechanics, degradation rate, drug release kinetics, and prebiotic activity. Development of such models would move the field beyond empirical trial-and-error modification and enable rational design of tailored derivatives.

Interindividual variability in colonic degradation represents another key uncertainty. KGM’s colon-targeting mechanism relies on microbiota-derived β-mannanase, but degradation rates vary widely across individuals depending on gut microbiome composition. Few studies have examined how disease states such as IBD or obesity, or interventions such as antibiotic use, alter KGM degradation in vivo, and this variability undermines the reliability of KGM-based delivery systems.

Human pharmacokinetic and long-term toxicological data remain scarce. Most studies to date have been conducted in rodent models, and there is limited information on the systemic absorption, tissue distribution, and long-term safety of KGM degradation fragments or chemically modified derivatives in humans. Toxicological characterization of functionalized KGM is particularly incomplete: carboxymethylated, oxidized, sulfated, and grafted derivatives lack systematic evaluation of metabolic fate, immunogenicity, and chronic toxicity, representing a major regulatory barrier.

Industrial-scale production methods remain underdeveloped relative to laboratory-scale techniques. Advanced extraction and modification processes have been validated only at gram scale, and there is a need for continuous processing, solvent recovery, and immobilized enzyme technologies to reduce production costs and environmental impact, and to make KGM competitive with established commercial excipients.

### 8.2. A Convergent Roadmap for the Next Decade

Moving forward, three convergent research directions could address these gaps and unlock the full potential of KGM.

Machine learning models trained on existing experimental data, combined with molecular dynamics simulations, could predict optimal Mw/DS/pattern combinations for specific applications. Active learning loops—in which model predictions guide synthesis, and experimental results iteratively improve the model—could substantially accelerate development of next-generation KGM derivatives with tailored properties, moving beyond the current trial-and-error paradigm.

As microbiome diagnostics become clinically available, KGM-based delivery systems could be designed to match individual patients’ microbiota. Combining KGM with defined primary degrader probiotics (e.g., *B. ovatus*) could reduce interindividual variability and improve the reliability of colon-targeted therapy. In functional foods, KGM could be formulated as part of personalized fiber blends matched to individual enterotypes, representing a new paradigm in precision nutrition.

Current KGM production generates substantial waste streams, including starch, protein, and fiber from tuber processing. Integrated biorefinery concepts that co-produce KGM, konjac glucomannan oligosaccharides (prebiotics), and protein isolates from the same feedstock could improve economics while reducing environmental impact. Emerging applications in flexible electronics (porous ZnO templated by KGM for gas sensing), environmental remediation (heavy metal adsorption), and 3D bioprinting represent additional high-value outlets that could transform KGM from a commodity food gum to a high-value specialty biopolymer [45].

## 9. Conclusions

This review has critically examined the molecular architecture, modification strategies, pharmaceutical applications, and health benefits of KGM, with a focus on structure–function relationships and translational challenges. Unique rheological and biological properties of KGM are governed by hierarchical structural features—acetylation pattern, molecular weight, and chain conformation—yet the field remains constrained by inconsistent characterization standards, unresolved structural controversies (notably regarding branching frequency and acetyl group distribution), and a predominantly empirical approach to chemical modification that lacks predictive design rules. While KGM has demonstrated considerable promise as a platform for colon-targeted drug delivery, probiotic encapsulation, wound healing, and metabolic health benefits—with multi-omics studies beginning to unravel mechanistic underpinnings—translation from laboratory proof-of-concept to clinical and commercial products remains slow, hampered by batch-to-batch variability, interindividual differences in colonic degradation, and regulatory hurdles for modified derivatives. Addressing these gaps through convergent, interdisciplinary efforts—combining advanced structural characterization, quantitative structure–activity modeling, and rigorous clinical validation—will be essential to fully realize the potential of KGM as a distinctive, sustainable biopolymer with unique advantages in targeted delivery, precision nutrition, and biomedical engineering.

## Figures and Tables

**Table 1:** Comparative performance of KGM against competing natural biopolymers for food and biomedical applications.

Property	KGM	Alginate	Chitosan	Xanthan	Gellan
Charge	Neutral [1]	Anionic [14]	Cationic [15]	Anionic [10]	Anionic [16]
Gelation type	Alkali-induced thermoirreversible [11]	Ionotropic (Ca^2+^) [14]	pH/ionic [14]	Thermoreversible [13]	Ion-induced thermoreversible [16]
Swelling ratio	50–100 [14]	20–40 [17]	10–30 [14]	5–15 [13]	10–25 [16]
Colonic degradation	Yes (β-mannanase) [2]	Partial [14]	Yes [14]	Limited [13]	Limited [16]
GRAS status	Yes [18]	Yes [14]	Limited [15]	Yes [13]	Yes [16]
Key limitation	Mechanical weakness [19]	Ion sensitivity [14]	Solubility at neutral pH [15]	High cost [13]	Brittle gels [16]

## Data Availability

No new data were created or analyzed in this study. Data sharing is not applicable to this article.
